# Paclitaxel-Loaded Nanosponges Inhibit Growth and Angiogenesis in Melanoma Cell Models

**DOI:** 10.3389/fphar.2019.00776

**Published:** 2019-07-12

**Authors:** Nausicaa Clemente, Monica Argenziano, Casimiro Luca Gigliotti, Benedetta Ferrara, Elena Boggio, Annalisa Chiocchetti, Fabrizio Caldera, Francesco Trotta, Elisa Benetti, Laura Annaratone, Simone Ribero, Stefania Pizzimenti, Giuseppina Barrera, Umberto Dianzani, Roberta Cavalli, Chiara Dianzani

**Affiliations:** ^1^Department of Health Sciences and Interdisciplinary Research Center of Autoimmune Diseases (IRCAD), UPO, Novara, Italy; ^2^Dipartimento di Scienza e Tecnologia del Farmaco, University of Torino, Torino, Italy; ^3^Department of Chemistry, University of Torino, Torino, Italy; ^4^Department of Medical Sciences, University of Torino, Torino, Italy; ^5^Department of Clinical and Biological Sciences, University of Torino, Torino, Italy

**Keywords:** paclitaxel, melanoma, angiogenesis, tumor growth, mouse model, nanosponges

## Abstract

This study investigated the effects of free paclitaxel (PTX) and PTX-loaded in pyromellitic nanosponges (PTX-PNS) in reducing *in vitro* and *in vivo* melanoma cell growth and invasivity, and in inhibiting angiogenesis. To test the response of cells to the two PTX formulations, the cell viability was evaluated by MTT assay in seven continuous cell lines, in primary melanoma cells, both in 2D and 3D cultures, and in human umbilical vein endothelial cells (HUVECs) after exposure to different concentrations of PTX or PTX-PNS. Cell motility was assessed by a scratch assay or Boyden chamber assay, evaluating cell migration in presence or absence of diverse concentrations of PTX or PTX-PNS. The effect of PTX and PTX-PNS on angiogenesis was evaluated as endothelial tube formation assay, a test able to estimate the formation of three-dimensional vessels *in vitro*. To assess the anticancer effect of PTX and PTX-PNS in *in vivo* experiments, the two drug formulations were tested in a melanoma mouse model obtained by B16-BL6 cell implantation in C57/BL6 mice. Results obtained were as follows: 1) MTT analysis revealed that cell proliferation was more affected by PTX-PNS than by PTX in all tested cell lines, in both 2D and 3D cultures; 2) the analysis of the cell migration showed that PTX-PNS acted at very lower concentrations than PTX; 3) tube formation assay showed that PTX-PNS were more effective in inhibiting tube formation than free PTX; and 4) *in vivo* experiments demonstrated that tumor weights, volumes, and growth were significantly reduced by PTX-PNS treatment with respect to PTX; the angiogenesis and the cell proliferation, detected in the tumor samples with CD31 and Ki-67 antibodies, respectively, indicated that, in the PTX-PNS-treated tumors, the tube formation was inhibited, and a low amount of proliferating cells was present. Taken together, our data demonstrated that our new PTX nanoformulation can respond to some important issues related to PTX treatment, lowering the anti-tumor effective doses and increasing the effectiveness in inhibiting melanoma growth *in vivo*.

## Introduction

Melanomas are a heterogeneous group of aggressive and highly metastatic tumors (Radovic et al., 2012), representing the deadliest form of skin cancer. Nearly half of patients with metastatic melanomas harbor a valine–glutamine substitution in codon 600 of the serine/threonine kinase BRAF (BRAFV600 mutation) (Davies et al., 2002). BRAF inhibitors (BRAFi) target selectively the BRAF V600E/K genetic alteration and are widely used to treat melanoma patients harboring BRAFV600 mutation. Treatment with BRAFi results in high response rates. However, responses are short-lived, with a median time to progression of 5.1–8.8 months (Flaherty et al., 2010; Robert et al., 2015). The addition of a MEK inhibitor to a BRAFi extends the median duration of response from 5.6 to 9.5 months (Long et al., 2017).

Similar results have been observed in patients treated with anti–PD-1 (tumor programmed death ligand 1) monotherapy or a combination of anti–PD-1 and anti–CTLA-4 agents. A recent overall survival (OS) analysis of the phase 3 KEYNOTE-006 trial showed a 33-month OS rate of 50% in patients receiving pembrolizumab monotherapy, an anti-PD-1 monoclonal antibody (Robert et al., 2017). Despite the efficacy of BRAF-targeted and PD-1-related immune therapies in treating metastatic melanoma, a significant number of patients exhibit resistance. Although chemotherapeutic drugs, including dacarbazine, cisplatin, and paclitaxel (PTX), have been used, alone or in combination, without significant survival rate improvement (Bhatia et al., 2009), some patients with metastatic melanoma present remarkable responses to chemotherapeutic agents, even in the absence of a response to modern targeted therapies and immunotherapies (Simon et al., 2017).

PTX was originally isolated from the bark of the Pacific yew tree, *Taxus brevifolia*, and phase II clinical trials suggested that it had clinical activity in melanoma (Walker et al., 2005). In addition to the microtubule-stabilizer function and the induction of cytotoxicity, PTX has been found to induce immunogenic cell death, which results in augmented CD8+ T cell priming and cytotoxic activity (Song et al., 2017), regulating the immunosuppressive microenvironment in tumor (Pfannenstiel et al., 2010). However, free PTX showed non-selective distribution and poor water solubility (less than 0.3 μg/ml) (Konno et al., 2003). The common PTX formulation approved consists of Cremophor EL^®^ and ethanol solution, but these solvents present severe side effects. To overcome these limitations, PTX has been embedded in drug nanotherapeutics, including albumin and polymeric micelle nanoparticles to treat several types of tumors (Sofias et al., 2017). These nanoformulations can reduce serious adverse effects of PTX, like allergic reactions, nephrotoxicity, and neurotoxicity, but some of them showed insufficient solubilizing capacity and poor stability (Mittal et al., 2018). For instance, Abraxane^®^, human serum albumin-bound PTX nanoparticle, approved by FDA in 2005, reduced off-targeted side effects and improved antitumor efficacy, but after i.v. administration, Abraxane^®^ rapidly dissociates into the individual constituents. Moreover, the pharmacokinetics and the biodistribution of PTX are not improved (Chen et al., 2018). Thus, identification of non-toxic formulations capable to deliver PTX to the target site and release it in a sustained manner is needed to avoid the nonspecific biodistribution and to prevent the toxicity due to excessive dose of the drug. The novel pyromellitic nanosponges (PNS) showed the capability to act as PTX nanocarrier able to store and release it slowly and in a prolonged manner.

In this paper, we compare the effectiveness of free PTX and PTX-loaded PNS (PTX-PNS) in inhibiting *in vitro* and *in vivo* melanoma cell growth and invasiveness and in inhibiting angiogenesis.

## Materials and Methods

### Preparation of PTX-Loaded Pyromellitic Nanosponges

PNS were synthetized by reacting β-cyclodextrin with pyromellitic anhydride as crosslinking agent at 1:4 molar ratio (CD/cross-linker). To transform the coarse PNS powder into a nanoformulation suitable for intravenous administration, a top down method was tuned. PNS were suspended in saline solution (NaCl 0.9% w/v) at the concentration of 10 mg/ml and homogenized using a high-shear homogenizer Ultra-Turrax (10 min, 24,000 rpm). Then, a high-pressure homogenization (HPH) step was performed to reduce the PNS size, using a high-pressure homogenizer (EmulsiFlex C5, Avastin, 90 min, 500 bar). The PNS nanosuspension was then purified by dialysis (membrane cutoff 12,000 Da). PTX-PNS were obtained by adding PTX solubilized in 50 µl of isopropanol to the blank PNS nanosuspension. The mixture was stirred at room temperature for 24 h.

### Physico-Chemical Characterization of PTX-Loaded Pyromellitic Nanosponges

Size, polydispersity index, and zeta potential values of blank and PTX-PNS were measured by dynamic light scattering using a 90 Plus particle sizer (Brookhaven Instruments Corporation, USA). The measurements were performed using diluted PNS samples at a fixed angle of 90° and at a temperature of 25 °C. For zeta potential determination, the samples were placed in the electrophoretic cell, where an electric field of about 15 V/cm was applied.

The PNS morphology was evaluated by transmission electron microscopy (TEM) analysis, using a Philips CM 10 transmission electron microscope. PNS samples were sprayed on Formvar-coated copper grid and air-dried before observation.

### Paclitaxel High Performance Liquid Chromatography (HPLC) Quantitative Determination

PTX quantitative determination was carried out by HPLC analysis using a pump (Perkin Elmer Pump 250B, Waltham, MA) equipped with a spectrophotometer detector (Flexar UV/Vis LC spectrophotometer detector, Perkin Elmer, Waltham, MA). A reverse phase Agilent TC C18 column (150 cm × 4.6 mm, pore size 5 μm; Agilent Technologies, Santa Clara, CA, USA) was used. The column was eluted with acetonitrile/water (60:40) at a flow rate of 1 ml/min. PTX was detected at 227 nm with a UV/vis detector. The drug concentration was calculated using the external standard method from a standard calibration curve.

### *In vitro* Release Studies

The release kinetics of PTX from PTX-PNS was *in vitro* evaluated. *In vitro* drug release studies were conducted in a multi-compartment rotating cell, comprising a donor chamber separated from the receiving phase by a cellulose membrane (Spectrapore, cut-off = 12,000 Da); 1 ml of PTX-PNS was placed in the donor chamber. The receiving chamber contained 1 ml of phosphate buffer 0.05 M (pH 7.4 or pH 5.5) added with 10% ethanol to assure drug solubility. The receiving phase was withdrawn at regular intervals and completely replaced with the same amount of fresh buffer to maintain sink conditions. The concentration of PTX in the withdrawn samples was detected by HPLC.

### Cell Cultures and Treatments

The following human melanoma cell lines were used: A375 from the American Type Culture Collection (ATCC; Manassas VA), M14, JR8, RPMI7932, PCF-2, and LM from Dr. Pistoia (Gaslini Institute, Genoa, Italy). The mouse melanoma B16-BL6 cell line was obtained from RIKEN, Saitama, Japan (RIKEN is Japan’s largest comprehensive research institution renowned for high-quality research in a diverse range of scientific disciplines). These cells were cultured in RPMI1640 medium, except A375 that were cultured in DMEM. Both media were supplemented with 10% fetal bovine serum (FBS), 100 units/ml penicillin, and 100 μg/ml streptomycin in a 5% CO_2_, 37 °C incubator. Human umbilical vein endothelial cells (HUVECs) were isolated from human umbilical veins by trypsin treatment (1%) and cultured in M199 medium with the addition of 20% FCS, 100 U/ml penicillin, 100 μg/ml streptomycin, 5 UI/ml heparin, 12 μg/ml bovine brain extract, and 200 mM glutamine. HUVEC were grown to confluence in flasks and used at the 2nd–5th passages. Use of HUVEC was approved by the Ethics Committee of the “Presidio Ospedaliero Martini” of Turin and conducted in accordance with the Declaration of Helsinki. Written informed consent was obtained from patients.

PTX was purchased from Bristol-Myers Squibb (Chester, UK).

### Isolation and Characterization of Primary Melanoma Cells

The primary melanoma cell line (PMel) was isolated from a 77-year-old Caucasian male patient with a superficial spreading melanoma in vertical growth phase, showing infiltration of the papillary dermis and cutaneous ulceration without metastasis. The study was approved by the Committee for human Biospecimen Utilization (ChBU—Department of Medical Sciences, University of Turin). Written informed consent was obtained from the patient for tissue to be used in research.

The tissue sample, used for the primary cell culture establishment, was collected from the “left-over tissue” (residual tissue not used for diagnostic and therapeutic purposes) at the Department of Medical Sciences, Pathology Unit, University of Torino (Italy), in sterile tubes containing 10 ml of RPMI serum free medium, supplemented with 1% penicillin-streptomycin-fungizone. Primary cell culture isolation was performed as described by Annaratone et al. (2013) with minor modifications. Briefly, the tissue sample was washed three times with the same medium, then finely minced by surgical blades into approximately 1×1 mm fragments which were incubated at 37 °C with collagenase type IV (1 mg/ml; 1∶1 RPMI, final volume 10 ml), for 3–5 h until complete disaggregation of fragments was obtained. Digested tissue samples were shaken vigorously by hand to disaggregate possible residual large clumps. Collagenase activity was blocked by addition of 10 ml of RPMI with 10% FBS. After centrifugation at 800 rcf for 6 min, the cell pellets were re-suspended in complete culture medium. The final cell suspension was seeded in petri dishes as passage 0 and kept in a humidified incubator with 5% CO_2_ at 37 °C in DMEM-F12 medium supplemented with 10% FBS, 10 ng/ml human epidermal growth factor (EGF), 5 mg/ml insulin, 400 ng/ml hydrocortisone, 1% L-glutamine, and 1% penicillin-streptomycin-fungizone (Sigma-Aldrich). Culture medium was changed first at the time of cell attachment and, subsequently, three times a week. After three passages, the cells were characterized for two specific melanoma markers, S100 and HMB45 by immunocytochemistry (ICC). ICC was performed by using an automated slide processing platform (Ventana BenchMark XT Autostainer, Ventana Medical Systems, Tucson, AZ, USA). The anti-S00 and the anti-HMB45 antibodies were purchased from DAKO and used following the manufacturer’s instructions (Milan, Italy). Both markers were positive (data not show), confirming the isolation of melanoma-type cells. PMel cells were maintained in standard 2D cell cultures, in DMEM-F12 medium supplemented as described above.

### Spheroid Formation of Primary Melanoma (PMeI) Cells

PMel cells, cultured in standard 2D condition, were dissociated with trypsin-EDTA into single-cell suspensions. The cells were then seeded on ultra-low attachment (ULA) 96-well flat-bottom plates (Sigma). Optimal seeding densities were established such that melanoma spheroids for both primary cell lines testes fell within a size range of 200 to 500 µm in diameter on day 8, considering appropriate for initiating experimental studies. Representative images of PMel tumor spheroids obtained on day 8, starting from 1×10^4^ cells/well, are showed in the **Supplementary data, Fig. S1**

### MTT Assay

The toxic effect of PTX or PTX-PNS was determined through the 3-(4,5-dimethylthiazol-2-yl)-2,5-diphenyltetrazolium bromide (MTT) assay as previously described (Ciamporcero et al., 2018). This colorimetric assay may be interpreted as a measure of both cell viability and cell proliferation (Sylvester, 2011). Cells were seeded (0.8 – 1.5 × 10^3^ cells/well) in 100 μl of serum-supplemented medium and treated with different concentrations of PTX and PTX-PNS. Untreated cells or cells treated with the empty PNS were used as control. After 72 h, the drug was removed and MTT assay was performed. The optical density (OD) of treated and untreated cells was determined at a wavelength of 570 nm with a microplate reader after 4 h of incubation. Controls were normalized to 100%, and the readings from treated cells were expressed as % of viability inhibition. Eight replicates were used to determine each data point, and five different experiments were performed.

### WST-1 Assay

The cytotoxic effect of PTX or PTX-PNS on PMel spheroids was determined by using the 2-(4-iodophenyl)-3-(4-nitrophenyl)-5-(2,4-disulfophenyl)-2H-tetrazolium (WST-1) reagent (Roche, Italy). Cells were seeded (0.8 – 1.5 ×10^3^ cells/well) in 100 μl of serum-supplemented medium and treated with different concentrations of PTX, PTX-PNS, or PNS. After 72 h, the drug was removed and the WST-1 assay was performed. The OD of treated and untreated cells was determined at a wavelength of 450 nm with a microplate reader after 4 h of incubation. Controls were normalized to 100%, and the readings from treated cells were expressed as % of viability inhibition. Eight replicates were used to determine each data point, and five different experiments were performed.

### Crystal Violet Assay

For the quantitative determination of cells adhering to the plate after the 6 h treatment with different concentrations of PTX, PTX-PNS, or PNS, the violet crystal test was used. The violet crystal is a water-soluble dye with affinity for neutral pH DNA, soluble at acidic pH. The cells were washed after treatment, fixed, and stained with crystal violet-methanol. After careful washing, acetic acid was added and the reading was made with a spectrophotometer at 595 nm. The controls were normalized to 100%, and the readings from treated cells were expressed as % of viability inhibition. Four replicates were used to determine each data point, and five different experiments were performed.

### Cell Motility Assays

In the wound-healing assay, after starvation for 24  h in serum-free medium, HUVECs were plated onto six-well plates (10^6^ cell/well) and grown to confluence. Cell monolayers were wounded by scratching with a pipette tip along the diameter of the well, and they were washed twice with serum-free medium before their incubation with diverse concentrations of PTX, PTX-PNS, or PNS. Drug concentrations that were not cytotoxic were used for this assay. In order to monitor cell movement into the wounded area, five fields of each wound were photographed immediately after the scratch (T0) and after 24 h (Dianzani et al., 2014). The endpoint of the assay was measured by calculating the reduction in the width of the wound after 24 h and compared to T0, which is set at 100%. The area of wound healing was calculated by using the ImageJ software (Schneider et al., 2012).

In the Boyden chamber (BD Biosciences, San Jose, CA) invasion assay, cells (2 × 10^3^) were plated onto the apical side of 50 μg/ml Matrigel-coated filters (8.2-mm diameter and 0.5-μm pore size; Neuro Probe, Inc.; BIOMAP snc, Milan, Italy) in serum-free medium with or without increasing concentration of PTX, PTX-PNS, or PNS. Nontoxic drug concentrations were used for this assay. Medium containing VEGF-α (10 ng/ml) was placed in the basolateral chamber as a chemo attractant for HUVEC and FCS 20% for melanoma cancer cells (A2058 and B16-BL6). After 6 h, cells on the apical side were wiped off with Q-tips. Cells on the bottom of the filter were stained with crystal violet, and all the fields were counted with an inverted microscope.

### Tube Forming Assay

Nontoxic drug concentrations were used for the tube formation assay. HUVECs were seeded onto 48-well plates (5×10^4^/well) previously coated with 75 μl of growth factor-reduced Matrigel, with or without increasing concentration of PTX, PTX-PNS, or PNS. The morphology of the capillary-like structures formed by the HUVECs was analyzed by an inverted microscope after 6 h of culture, and photographed with a digital camera. Tubule formation was analyzed with an imaging system (Image-Pro Plus Software for microimaging, Media Cybernetics, version 5.0, Bethesda, MD, USA). Tube formation was evaluated by counting the total number of tubes in three wells, and five different experiments were performed. The results were expressed as % inhibition of untreated control cell.

### *In vivo* Experiments

Eight-week-old female C57BL6/J mice (Charles River Laboratories, Wilmington, MA, USA) were injected subcutaneously (s.c.) with B16-BL6 cells (10^5^ cells/mouse). The mice were bred under pathogen-free conditions in the animal facility of the Department of Health Sciences (UPO, Novara, Italy). All experimental procedures were done according to Europeans Guidelines and our Institution’s ethics commission. After 7 days from the injections, when average tumor dimension reached 5 mm^3^, mice were randomized in a blind fashion into homogenous groups (5 mice per group) and assigned to different treatments. Free PTX or PTX-PNS dissolved in NaCl 0.9% were administrated by tail injection (100 μl/mouse) at the dose of 2.5 mg/kg, every 4 days, for four times. Control mice were injected with empty PNS dissolved in PBS. Treatment-related toxicity was determined by monitoring mouse weight weekly. The tumor size was measured with a caliper, and mice were sacrificed 4 days after the last injection. Euthanasia, collection of tumor samples, tumor weight, and volume determination were performed after 2 weeks from the beginning of treatments.

### Histology and Immunofluorescence Anti-CD31 and Anti-Ki-67 on Tumor Sections

Immediately after dissection, tumor samples were embedded in OCT compound (Killik, Bio Optica Milano SpA) and stored at −80 °C until use. Tumor tissues were cut with a cryostat (thickness 4–5 µm) and treated with 4% paraformaldehyde (Sigma-Aldrich) diluted in PBS for 5 min at room temperature to fix the sample on the glass slides. The samples were then blocked with 5% normal goat serum (R&D System) in PBS for 1 h in order to block nonspecific sites to which the primary antibody could bind. To detect CD31 and Ki-67 expression, the primary antibodies used were a polyclonal rabbit anti-CD31 (Abcam, Cambridge, UK) or a monoclonal mouse anti-human Ki-67 antigen (DAKO); both diluted 1:50 and were incubated over night at 4°C in a humid chamber. The secondary antibody used was an anti-rabbit Ig Alexa Fluor 488-conjugated (Thermo-Fisher), or an anti-mouse Ig Alexa Fluor 546-conjugated (Thermo-Fisher); both diluted 1:400, respectively. Then, the sections were stained with 0.5 mg/ml of the fluorescent dye 4,6-diamidino-2-phenylindole-dihydrochloride (DAPI, Sigma-Aldrich) for 5 min to highlight cell nuclei and then mounted using prolong anti-fade mounting medium (SlowFade AntiFADE Kit, Molecular Probes Invitrogen). The sections were then observed by a fluorescence microscope (Leica, Italy) and analyzed by Image Pro Plus Software for micro-imaging 5.0 (Media Cybernetics, version 5.0, Bethesda, MD, USA). Tumor microvessel density (MVD) was measured by evaluating the CD31-positive area; the numbers of positive cells for Ki-67 was calculated in the total tumor area per field upon slide scanning (Panoramic midi II, 3D HISTECH, Budapest, Hungary). Hematoxylin and eosin (Sigma Aldrich, Milan, Italy) staining was performed to assess morphological changes.

### Statistical Analysis

Data were expressed as means ± SD. Significance between experimental groups was determined by one-way ANOVA followed by the Bonferroni multiple comparison post-test using GraphPad InStat software (San Diego, CA, USA). Values of p ≤ 0.05 were considered significant.

## Results

### Physico-Chemical Characterization of PTX-PNS

The physico-chemical parameters of PNS before and after loading with PTX are reported in **Table 1**. The PNS nanoformulations showed average diameters of about 300 nm and a negative surface charge. The drug incorporation slightly affected the physico-chemical characteristics. The zeta potential value remained enough high to avoid aggregation phenomena.

**Table 1:** Physico-chemical characteristics of blank and PTX-loaded PNS formulations.

Formulation	Average diameter ± SD (nm)	Polydispersity index	Zeta potential ± SD (mV)
Blank PNS	307.2 ± 10.6	0.22 ± 0.01	−33.6 ± 4.5
PTX-PNS	316.8 ± 12.1	0.20 ± 0.02	−28.4 ± 3.7

**Figure 1:**
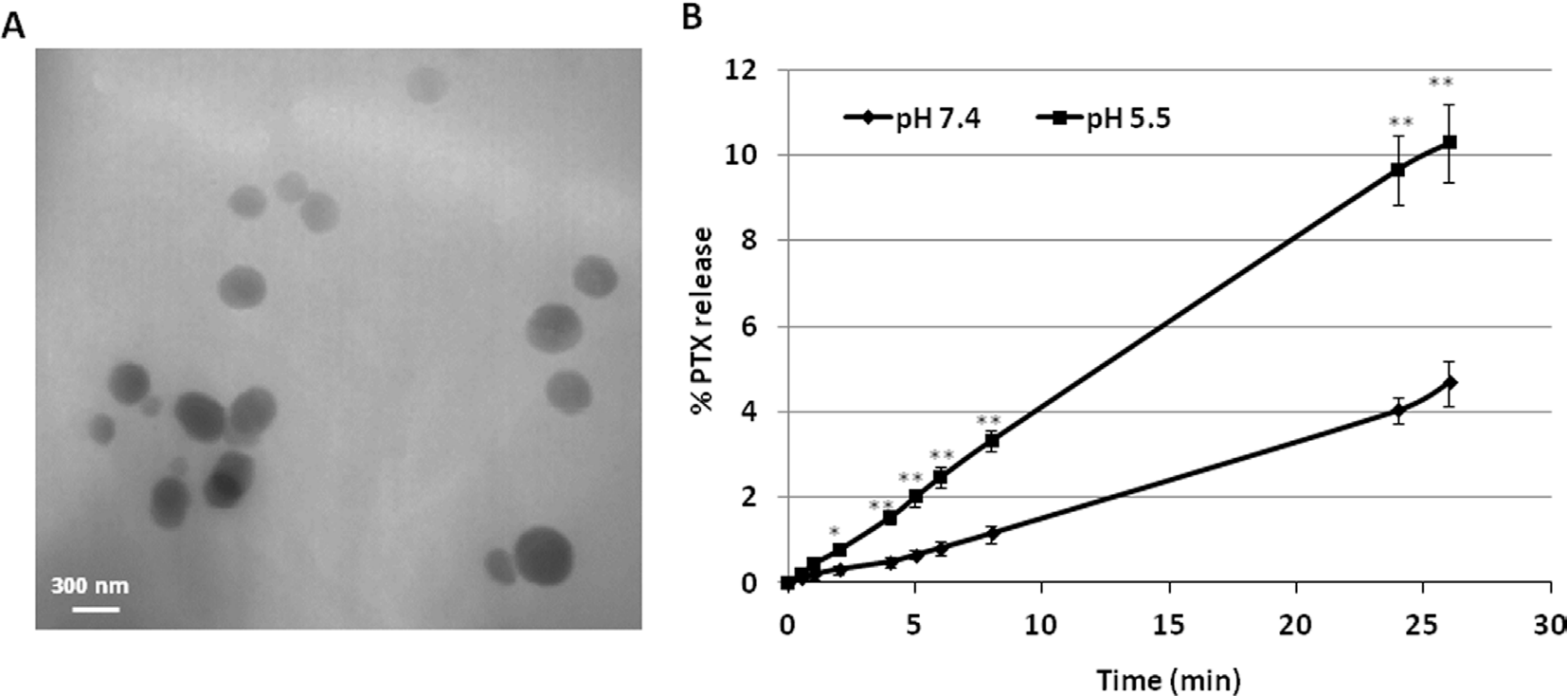
Characterization of PNS formulations. **(A)** TEM image of PTX-loaded PNS; **(B)** *In vitro* release kinetics of PTX from PTX- loaded PNS as a function of pH (pH 7.4 or pH 5.5).

TEM analysis showed the spherical morphology of PNS and confirmed their nanoscale sizes, due to the high pressure homogenization step. **Figure 1** (panel A) reports the TEM image of PTX-loaded PNS.

PNS were able to load PTX in a good extent, showing an encapsulation efficiency of about 96.5% and a loading capacity of about 8%.

The release profile of PTX from PTX-PNS was investigated *in vitro* at two pH values (**Figure 1**, panel B). Prolonged *in vitro* release kinetics was demonstrated, and no initial burst effect was observed. The sustained release of PTX from the PTX-PNS confirmed the drug incorporation in the PNS polymer matrix. The percentage of PTX released from the PNS was about 4% at pH 7.4 and 10% at pH 5.5 after 24 h, indicating an enhanced release kinetics at acidic pH.

### Effect of PTX and PTX-PNS on Cell Proliferation

**Figure 2:**
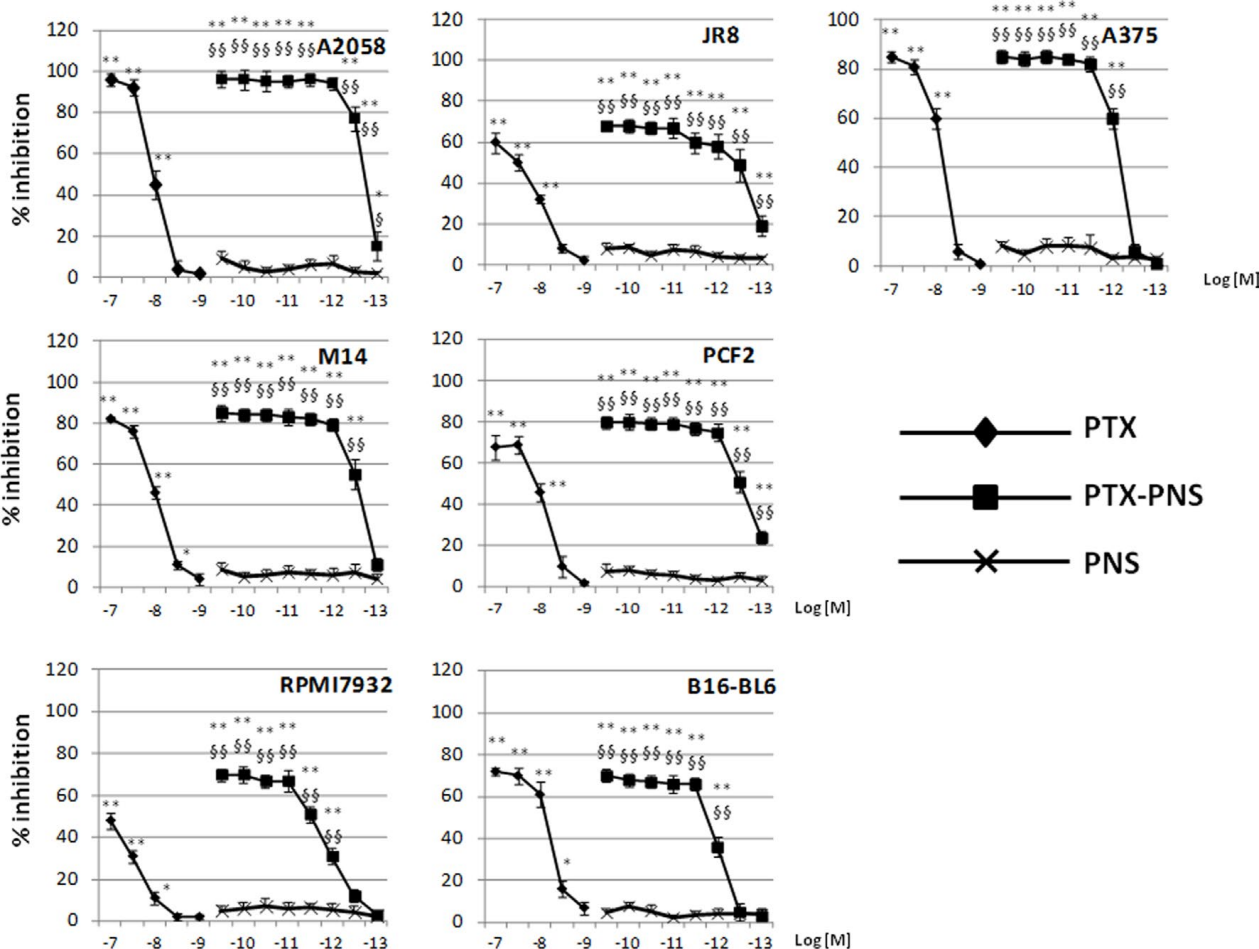
Inhibition of melanoma cell proliferation following PTX and PTX-PNS treatment. Cells were treated with increasing concentrations of PTX (from 10^-7^ to 10^-9^ M) or PTX-PNS (from 10^-10^ to 10^-13^ M) for 72 h. The results are expressed as % inhibition of control and are the mean ± SD of five separated experiments. **p < 0.01 *vs* control and PNS, *p < 0.05 *vs* control and PNS, ^§§^p < 0.01 *vs* PTX, ^§^p < 0.05 *vs* PTX.

To compare the response of cells to free PTX and PTX-PNS, we first analyzed cell viability after 72 h of exposure to different concentrations of PTX (from 10^-7^ to 10^-9^ M) and PTX-PNS or PNS (from 10^-10^ to 10^-13^ M). MTT analysis revealed that cells were more affected by PTX-PNS than by free PTX. The effective concentrations ranged from 10^-7^ to 10^-8^ M for the free PTX in all cell lines; from 3×10^-9^ to 10^-13^ M PTX-PNS in A2058, JR8, PCF2; from 3×10^-9^ to 3×10^-12^ M PTX-PNS in M14; and from 3×10^-9^ to 10^-12^ M PTX-PNS in A375, RPMI7932, and B16-BL6 (**Figure 2**). The empty PNS did not show any toxicity even at highest doses, and the MTT values were similar to those obtained in untreated cells.

### Effect PTX and PTX-PNS in Inhibiting Growth of 2D and 3D Cultures of Primary Melanoma Cells

**Figure 3:**
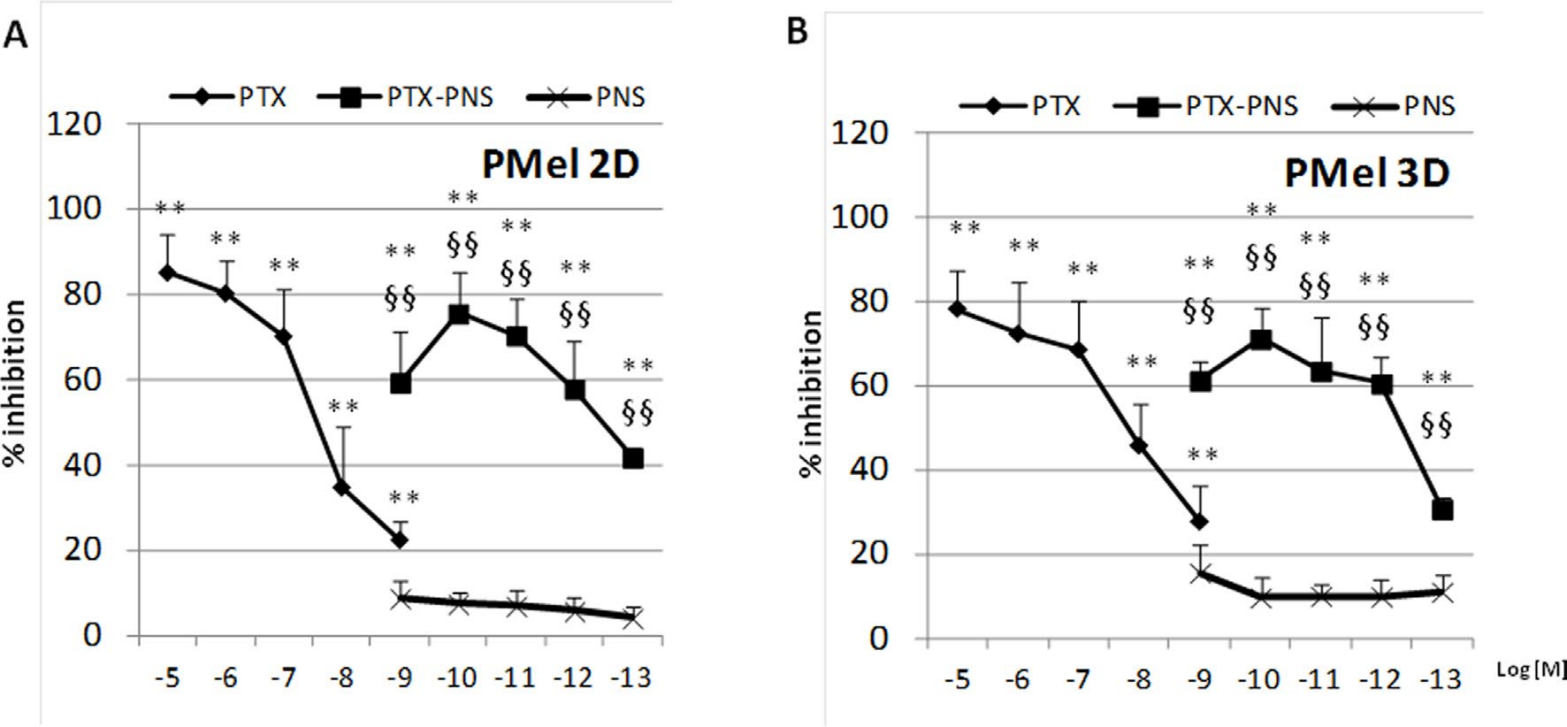
Inhibition of primary melanoma cell growth following PTX and PTX-PNS treatment. Primary melanoma cells **(A)** in 2D or **(B)** in 3D cultures were treated with increasing concentrations of PTX (from 10^-5^ to 10^-9^ M) or PTX-PNS (from 10^-9^ to 10^-13^ M) or PNS (10^-9^ M) for 72 h. The results are expressed as % inhibition of control and are the mean ± SD of five separated experiments. **p < 0.01 *vs* control and PNS, ^§§^p < 0.01 *vs* PTX.

In primary melanoma PMel cells, PTX and PTX-PNS showed an inhibitory activity of growth at different doses. PTX was effective at doses ranging from 10^-5^ to 10^-9^ M, whereas PTX-PNS was effective at doses ranging from 10^-9^ to 10^-13^ M. The empty PNS did not show any toxicity (**Figure 3A**). In 3D spheroids, PTX inhibited the growth at concentrations ranging from 10^-5^ to 10^-9^M, whereas PTX-PNS inhibited growth at concentrations ranging from 10^-9^ to 10^-13^ M (**Figure 3B**).

### Effect of PTX and PTX-PNS in Inhibiting Cell Migration

**Figure 4:**
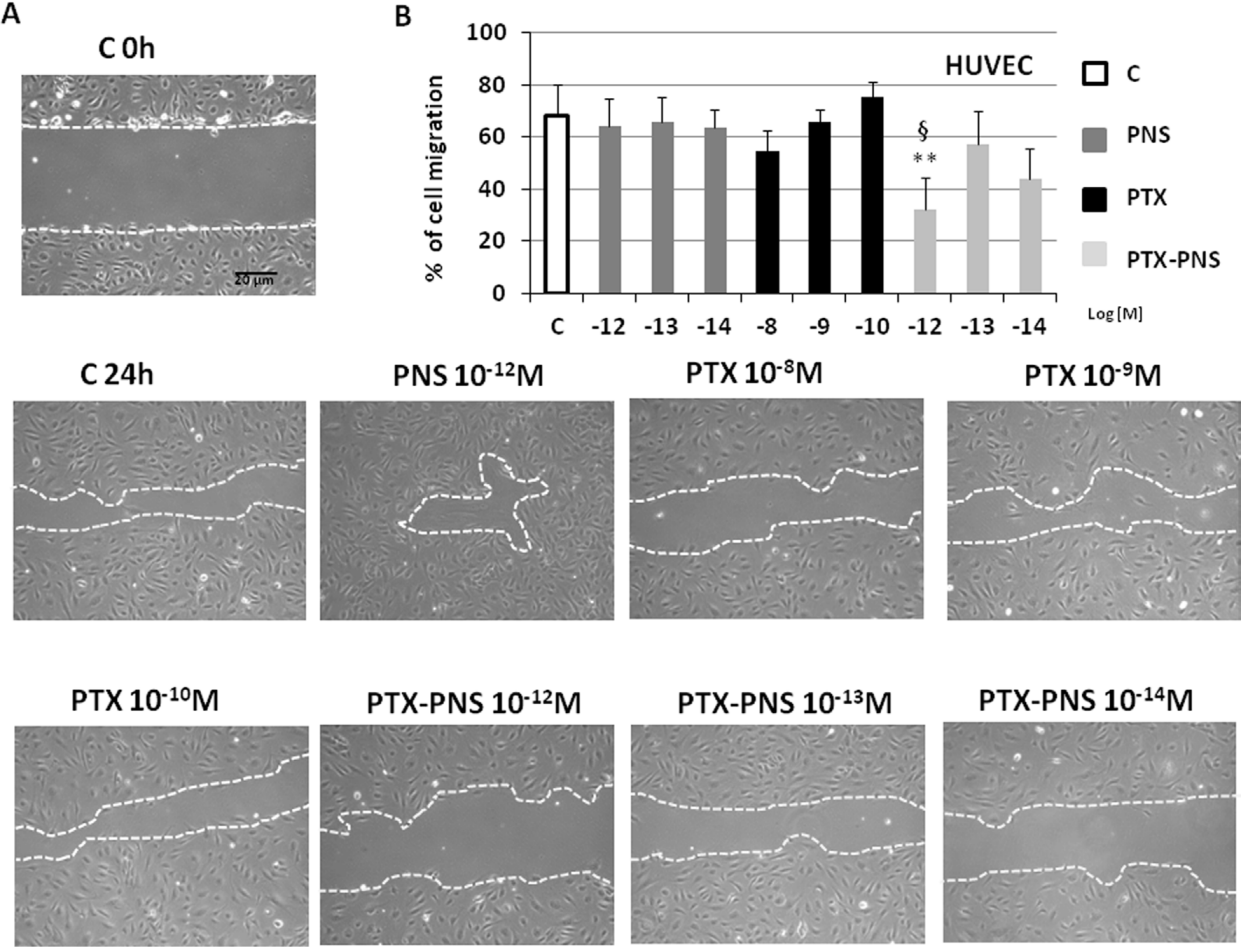
Wound-healing assay of HUVEC treated with different concentrations of PTX and PTX-PNS. A scratch was made through the HUVEC layer, and then, cells were cultured in the absence (C) or in presence of PTX (from 10^-8^ to 10^-10^ M) or PTX-PNS (from 10^-12^ to 10^-14^ M) or PNS (10^-12^ M) for 24 h. **(A)** Microphotographs of the wounded area were taken immediately after the scratch (0 h) and after 24 h, in order to monitor cell migration into the wounded area. **(B)** The graph shows mean ± SD (n = 5) of assay endpoints measured by calculating the reduction in the width of the wound after 24 h and compared to T0 which is set at 100%. The area of wound healing was calculated by using the ImageJ software. ^∗∗^p < 0.01 *vs* C, ^§^p < 0.05 *vs* PTX.

Tumor growth is favored by tumor angiogenesis, which is continuously activated in cancer resulting in the accumulation of immature and chaotic blood vessels. The acquisition of endothelial cell motility represents the first step of angiogenesis. In order to find the PTX and PTX-PNS nontoxic concentrations in HUVECs, which can be used in the migration test, MTT analysis was performed after 24 h. HUVECs were cultured in the presence and absence of titrated amounts of the different formulations. Results demonstrated that PTX concentration ranging from 10^-8^ to 10^-10^ M and PTX-PNS concentrations ranging from 10^-12^ to 10^-14^ M were nontoxic for HUVEC cells at 24 h (**Supplementary Data, Figure S2**). Thus, PTX concentrations in the range of 10^-8^–10^-10^ M and PTX-PNS concentrations from 10^-12^ to 10^-14^ M were chosen for the wound-healing migration test. Analysis of cells ability to migrate into the scratch showed that only PTX-PNS inhibited HUVEC migration at 10^-12^ M (**Figure 4A and B**), while PTX was unaffective.

**Figure 5:**
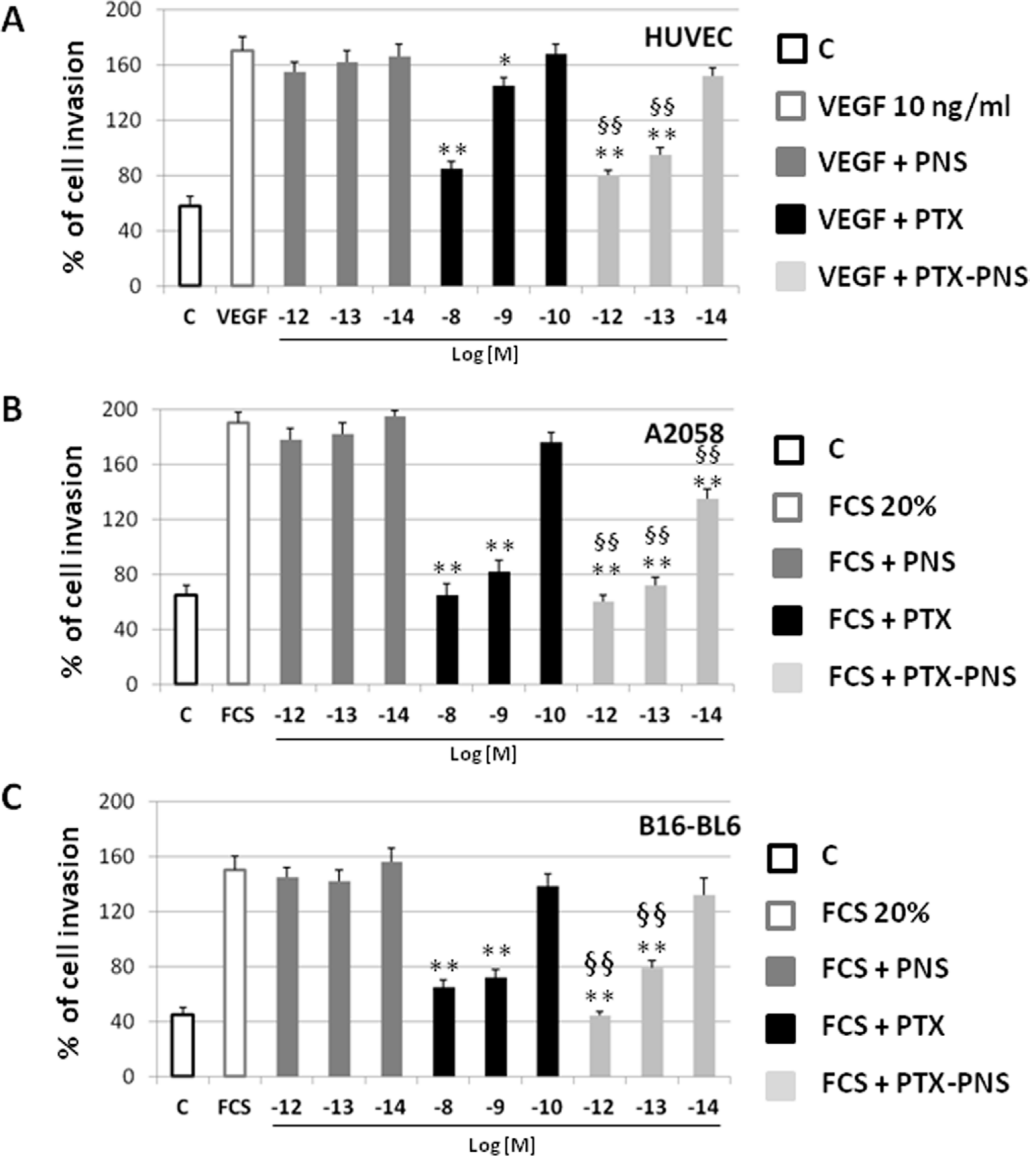
Effect of PTX and PTX-PNS on motility of HUVEC **(A)**, A2058 **(B)**, and B16-BL6 **(C)** assessed by Boyden chamber assay. HUVECs were plated onto the apical side of Matrigel-coated filters in the presence and absence of either PTX (from 10^-8^ to 10^-10^ M) or PTX-PNS (from 10^-12^ to 10^-14^ M). Medium containing VEGF-α (10 ng/ml) or FCS 20% was placed in the basolateral chamber as a chemoattractant for HUVECs or melanoma cell line, respectively. After 6 h, cells on the apical side were wiped off with Q-tips. Cells on the bottom of the filter were stained with crystal violet, and all counted with an inverted microscope. Data are expressed as mean ± SD (n = 5) of number of migrated cells. ^∗∗^p < 0.01, *vs* VEGF-α or FCS, ^§§^p < 0.01, *vs* PTX.

To confirm these results, cell motility was measured by using a Boyden chamber assay, assessing the capability of directional migration and invasion. In order to find the PTX and PTX-PNS concentrations that were not cytotoxic in HUVECs and melanoma cells, crystal violet assay was performed after 6-h treatments with titrated amounts of the diverse formulations. Results demonstrated that cell viability was not affected by any concentration of the drug formulations tested (**Supplementary data, Table S1**). The invasion experiments demonstrated that PTX and PTX-PNS inhibited HUVEC invasion in a concentration dependent-manner; PTX was active at 10^-8^–10^-9^ M, whereas PTX-PNS affected cell invasion at concentrations ranging from 10^-12^ to 10^-13^ M (**Figure 5A**). Similar results were obtained for human and mouse melanoma cell lines (**Figure 5B and C**). Representative images of crystal violet staining Matrigel-coated filters of the Boyden chambers were reported in **Supplementary Data, Figure S3**.

### PTX and PTX-PNS inhibit angiogenesis

**Figure 6:**
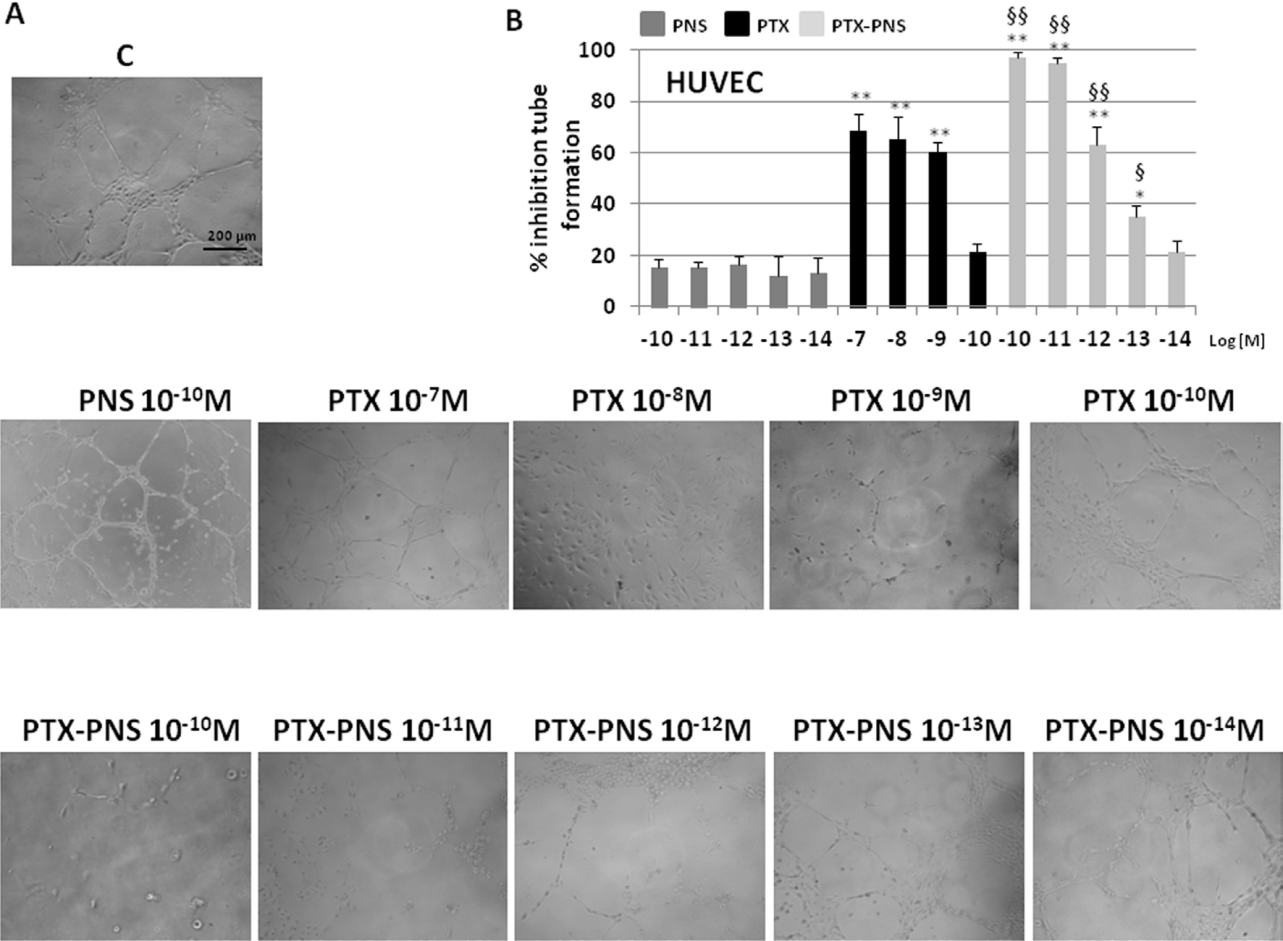
Tube formation assay of HUVEC treated with different concentrations of PTX or PTX-PNS. HUVEC were plated in the presence and absence of PTX (from 10^-7^ to 10^-10^ M) or PTX-PNS (from 10^-10^ to 10^-14^ M). **(A)** The morphology of the capillary-like structures formed by the HUVEC was analyzed by an inverted microscope after 6 h of culture, and photographed with a digital camera. **(B)** The graph shows the tube formation, evaluated by counting the total number of tubes in three wells of five different experiments. The results were expressed as % inhibition of untreated control cells. Data are expressed as mean ± SD. ^∗∗^p < 0.01, *vs*.VEGF-α or FCS, ^∗^p < 0.05, *vs* VEGF-α or FCS, ^§§^p < 0.01, *vs*. PTX, ^§^p < 0.05, *vs* PTX.

The effect of PTX and PTX-PNS on angiogenesis was evaluated in endothelial tube formation assay, which is able to estimate the formation of three-dimensional vessels *in vitro*. HUVECs were seeded onto 24-well plates (5×10^4^ cell/well) previously coated with 75 μl of growth factor-reduced Matrigel (BD Biosciences), in the absence or presence of nontoxic concentrations of PTX (10^-7^–10^-10^M) or PTX-PNS (10^-10^–10^-14^M) (**Supplementary Data, Table S1**). The morphology of capillary-like structures formed by HUVEC was analyzed 6 h after culturing. The results showed that PTX and PTX-PNS dose-dependently inhibited endothelial tube formation (**Figure 6A**). Quantification of the inhibition is shown in **Figure 6B**. PTX inhibited tube formation at the doses ranging from 10^-7^ to 10^-9^ M, whereas PTX-PNS were more effective inhibiting tube formation at the doses ranging from 10^-10^ to 10^-13^ M.

### PTX and PTX-PNS Anticancer Effect in Xenograft Tumor Model

**Figure 7:**
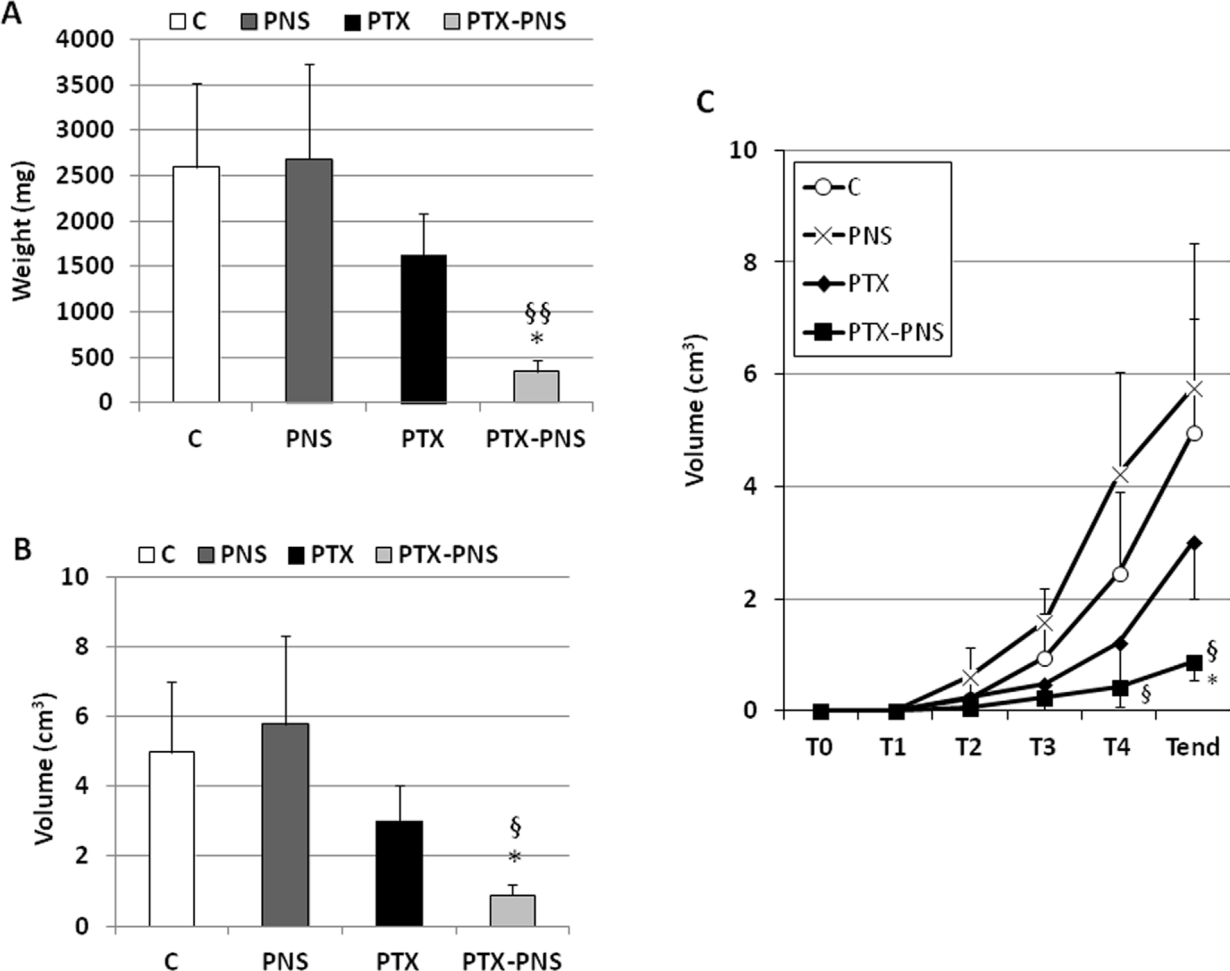
In vivo experiments on mouse melanoma model. C57BL6/J mice were injected subcutaneously with B16-BL6 cells (10^5^ cells/mouse). 7 days after the tumor injection, mice were treated every 4 days for 2 weeks by i.v. injection of PTX, PTX-PNS, and PNS (2.5 mg/kg, 100 µl/mouse) or the same volume of NaCl 0.9% as control (five mice/group). Mice were sacrificed at the end of the experiment. Graphs show **(A)** tumor weight (mg, mean ± SD), **(B)** tumor volume curves (cm^3^, mean ± SD), and **(C)** tumor growth (cm^3^, mean ± SD). Tumors were evaluated every 4 days, after the first treatment performed at T1 (i.e., when they were palpable). Data are expressed as mean ± SD. ^∗^p < 0.05, *vs* VEGF-α or FCS, ^§§^p < 0.01, *vs*. PTX, ^§^p < 0.05, *vs* PTX.

**Figure 8:**
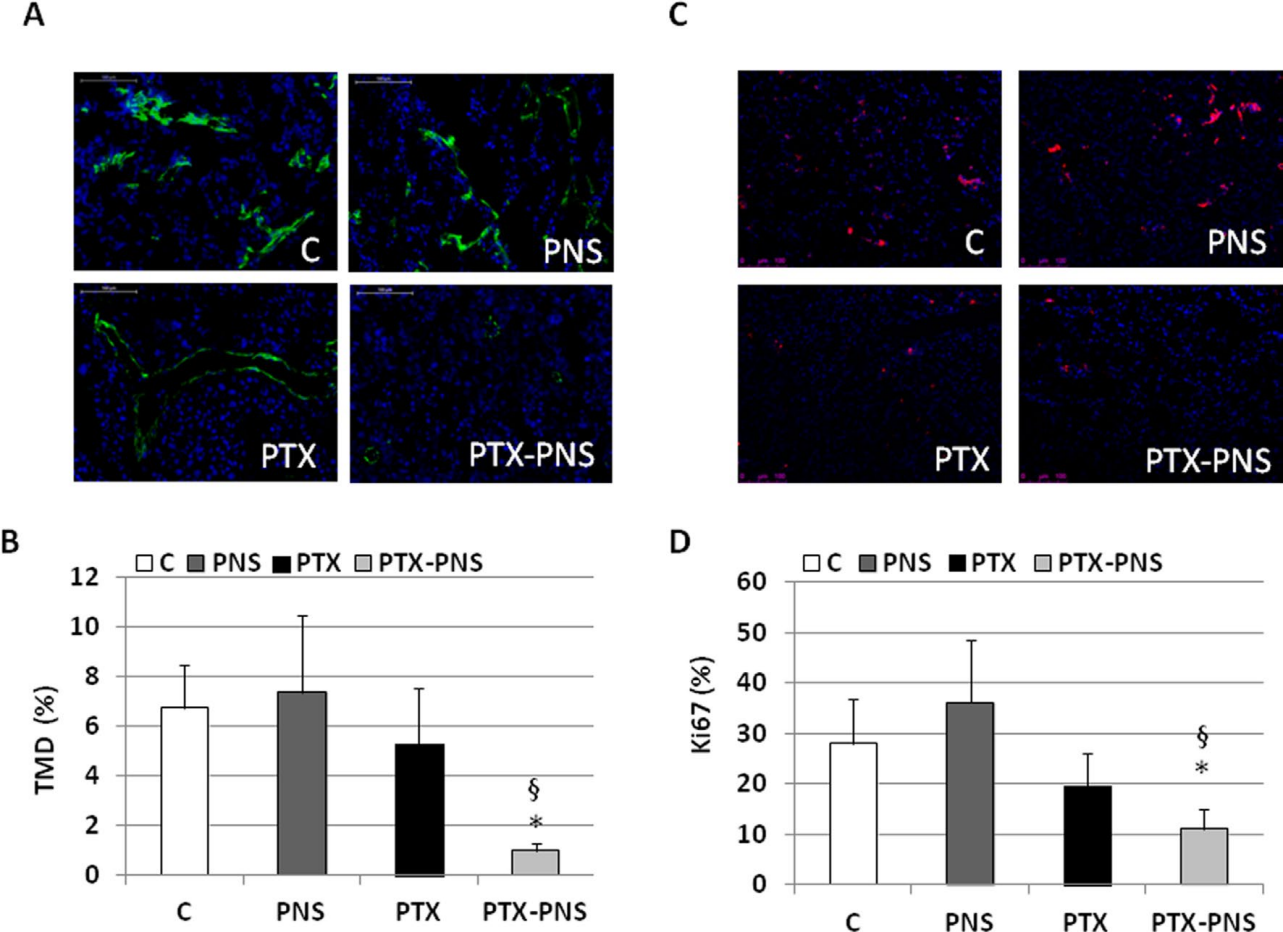
**(A)** Microphotographs of CD31 staining from a representative experiment (green; magnification 200x). **(B)** Tumor microvessel density (MVD) determined as the percentage of CD31-positive area on the tumor sections. Three randomly selected areas from three tumors from each group were analyzed. **(C)** Microphotographs of Ki-67 staining from a representative experiment (red; magnification 200x). **(D)** % of Ki-67-positive cells among tumor cells. Three randomly selected areas from three tumors from each group were analyzed. *p < 0.05 *vs* C and PNS, ^§^p < 0.05 *vs* PTX.

To assess the anticancer effect of PTX and PTX-PNS in *in vivo* experiments, we implanted B16-BL6 cells, in C57/BL6 mice, and we treated animals with the two drug formulations. Results showed that tumor weights (**Figure 7A**), volumes (**Figure 7B**), and growth (**Figure 7C**) were significantly reduced by PTX-PNS treatment compared to those detected in the mice treated with either PBS, empty PNS, or free PTX at the dose of 2.5 mg/kg. By contrast, PTX did not show any significant effect. Analysis of tumor vasculature was assessed by staining CD31 in the tumor sections and showed that vascular density (MVD) was significantly lower in the tumors from mice treated with PTX-PNS than in those treated with either PBS, empty PNS, or free PTX (**Figure 8A and B**). The number of positive cells for Ki-67 (**Figure 8C and D**) confirmed previous results. All treatments were well tolerated by the animals without significant weight loss in any group.

## Discussion

The use of nanodelivery systems offers some advantages that can improve the therapeutic efficacy of anticancer drugs. Indeed, nanoformulations could increase drug concentration at the tumor site, decreasing the total dose administered, and subsequently reducing the side effects (Duchene et al., 2016, Prasad et al., 2018). In particular, cyclodextrin-based nanosponges (NS) have been proposed for cancer nanotherapeutic development (Trotta et al., 2012, Trotta et al., 2014; Swaminathan et al., 2016). The use of NS as nanocarrier for PTX delivery was previously investigated. PTX was encapsulated in NS obtained by reacting CDs with diphenylcarbonate as cross-linker (Ansari et al., 2011).

The *in vivo* behavior of PTX-NS was studied after oral administration to rats, showing an increase of the drug oral bioavailability (Torne et al., 2010). Moreover, PTX showed the capability to be incorporated in a great extent in another type of NS, obtained using carbonyldiimidazole as crosslinking agent (Mognetti et al., 2012). Here, we evaluated the use of PNS as nanovehicle of PTX. Previously, PNS showed non-toxic effect *in vitro* and *in vivo* in acute and repeated dose toxicity studies (Shende et al., 2015). PNS were able to incorporate PTX, increasing its apparent aqueous solubility. Indeed, it has an extremely low aqueous solubility (less than 0.3 µg/) and it is currently dissolved in a mixture of Cremophor EL^®^ (polyoxyethyleneglycerol triricinoleate 35) and dehydrated ethanol (1:1 v/v) in the commercial intravenous dosage form. The PTX incorporation in the PNS nanostructure was confirmed by the slow and prolonged *in vitro* release kinetics of the drug from PTX-NS. Interestingly, results demonstrated that PTX-PNS inhibited melanoma cell growth more effectively than free PTX. The inhibitory activity on cell proliferation was effective on all of the melanoma cell lines used in this study, including a PMel. Moreover, the cytotoxicity of PTX-PNS was displayed at concentrations which were a thousand times lower than those displayed by free PTX. PTX-PNS significantly inhibited the proliferation of primary tumor cells in both 2D and 3D melanoma cell cultures with the same effectiveness. Results on melanoma spheroid 3D cultures were particularly relevant since 3D-cultured cells acquired morphological and cellular features which are more similar to solid tumors than 2D cultures. In particular, Ma et al. (2012) compared nanoparticle penetration properties of different culture systems and reported that 3D spheroids of HeLa cells displayed similar morphologic features of human solid tumors, including a resistance to chemotherapeutics that could not be observed in 2D cultures. In line with this observation, it has been suggested that 3D spheroids may be a useful simplified model of tumor tissue for *in vitro* testing of anticancer therapeutics (Edmondson et al., 2014; Huang et al., 2015).

The effectiveness of PTX-PNS in 3D cultures demonstrated that this nanoformulation is effective on a tumor-like environment mimicking several features of tumors involved in chemotherapy resistance such as three-dimensional architecture, cell–cell interaction, and hypoxia.

Cancer metastasis is associated with stimulation of cancer cell migration and invasion of the neighboring tissues. In line with previous results, PTX-PNS inhibited cell melanoma invasion at concentrations which were much lower than those displayed by free PTX.

Cancer progression is also associated with stimulation of tumor neoangiogenesis producing newly formed vessels to feed the tumor. This process involves endothelial cell migration and generation of tubule-like structures to form vessels. PTX can reduce endothelial cell migration at concentrations ranging from 10^-7^ to 10^-9^ M, according to previous reports showing taxane effects on cell migration (Ballestrem et al., 2000). However, also in this case, PTX-PNS was much more effective in inhibiting HUVEC migration and invasion than free PTX. A similar pattern in *in vitro* tubulogenesis of endothelial cells was observed, since the inhibitory effect of PTX was obtained at nanomolar concentrations, in line with previous data from Taraboletti et al. (2002). Intriguingly, PTX-PNS were able to inhibit tubulogenesis at lower concentrations, than free PTX.

Finally, we demonstrated that PTX-PNS was more effective than PTX in inhibiting the *in vivo* growth of melanoma cells in a mouse model also. Indeed, both the weight, the volume, and the growth of melanoma were significantly reduced in mice treated with PTX-PNS whereas no significant inhibition was obtained with the same dose of free PTX. The results on angiogenesis and proliferation rate of tumor cells *in vivo* are in agreement with the *in vitro* experiments since the microvessel density in the tumor and the percentage of Ki-67 positive cells was significantly decreased by treatment with PTX-PNS, whereas no significant effect was obtained upon treatment with free PTX.

Taken together, our data demonstrated that our new PTX nanoformulation can respond to some important issues related to PTX treatment, such as solubility and toxicity. The PTX incorporation in nanosponges might allow to lower the anti-tumor doses and increase its effectiveness in inhibiting melanoma cell model.

## Ethics Statement

All experimental procedures were done according to Europeans Guidelines and our institution’s ethics commission.

## Author Contributions

CD, RC, GB, FT, and UD conceived the project. BF, MA, NC, FC, CLG, EBo, LA, EBe, and SR performed the experiments. SP, AC, and CD analyzed the results. GB, SP, and CD wrote the first draft. All authors revised the manuscript.

## Conflict of Interest Statement

The authors declare that the research was conducted in the absence of any commercial or financial relationships that could be construed as a potential conflict of interest.

## Supplementary Material

Figure S1 Spheroids obtained from primary melanoma cell line PMel.

Figure S2 Effect of PTX and PTX-PNS treatments on HUVEC cell viability assessed by MTT assay at 24h.

Figure S3 HUVECs, A2058 and B16-BL6 cells photographed after the invasion test.

Table S1 Effect of PTX and PTX-PNS treatments on HUVEC, A2058, and B16-BL6 on cell viability assessed by crystal violet staining at 6h.
