# Supplementary figures and images for: Paclitaxel-Loaded Nanosponges Inhibit Growth and Angiogenesis in Melanoma Cell Models
Source: Front Pharmacol. 2019 Jul 12;10:776. doi: 10.3389/fphar.2019.00776 (PMC6639435; doi:10.3389/fphar.2019.00776)

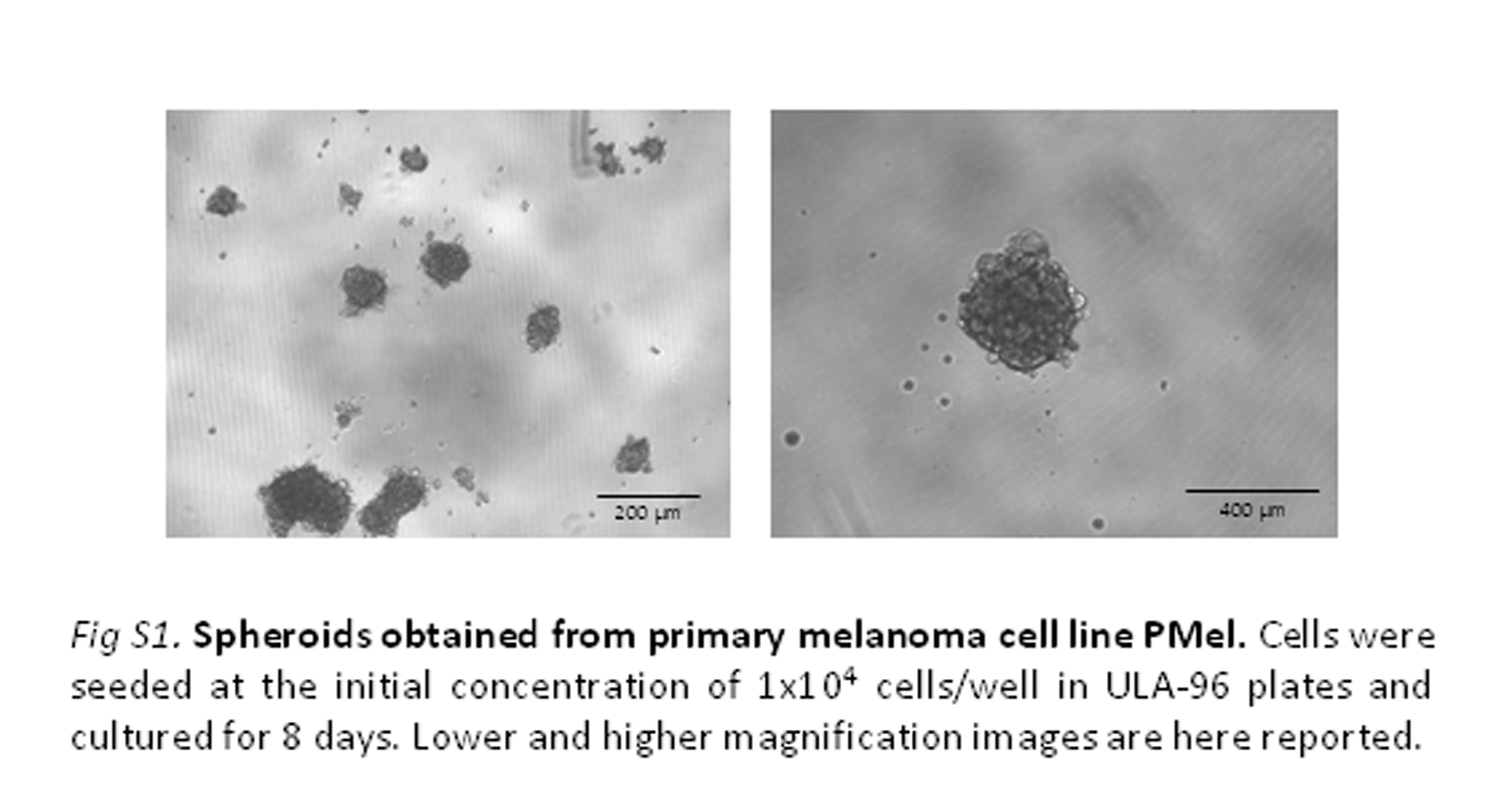

Supplement: Figure S1 — Spheroids obtained from primary melanoma cell line PMel. [file Image_1.tif]

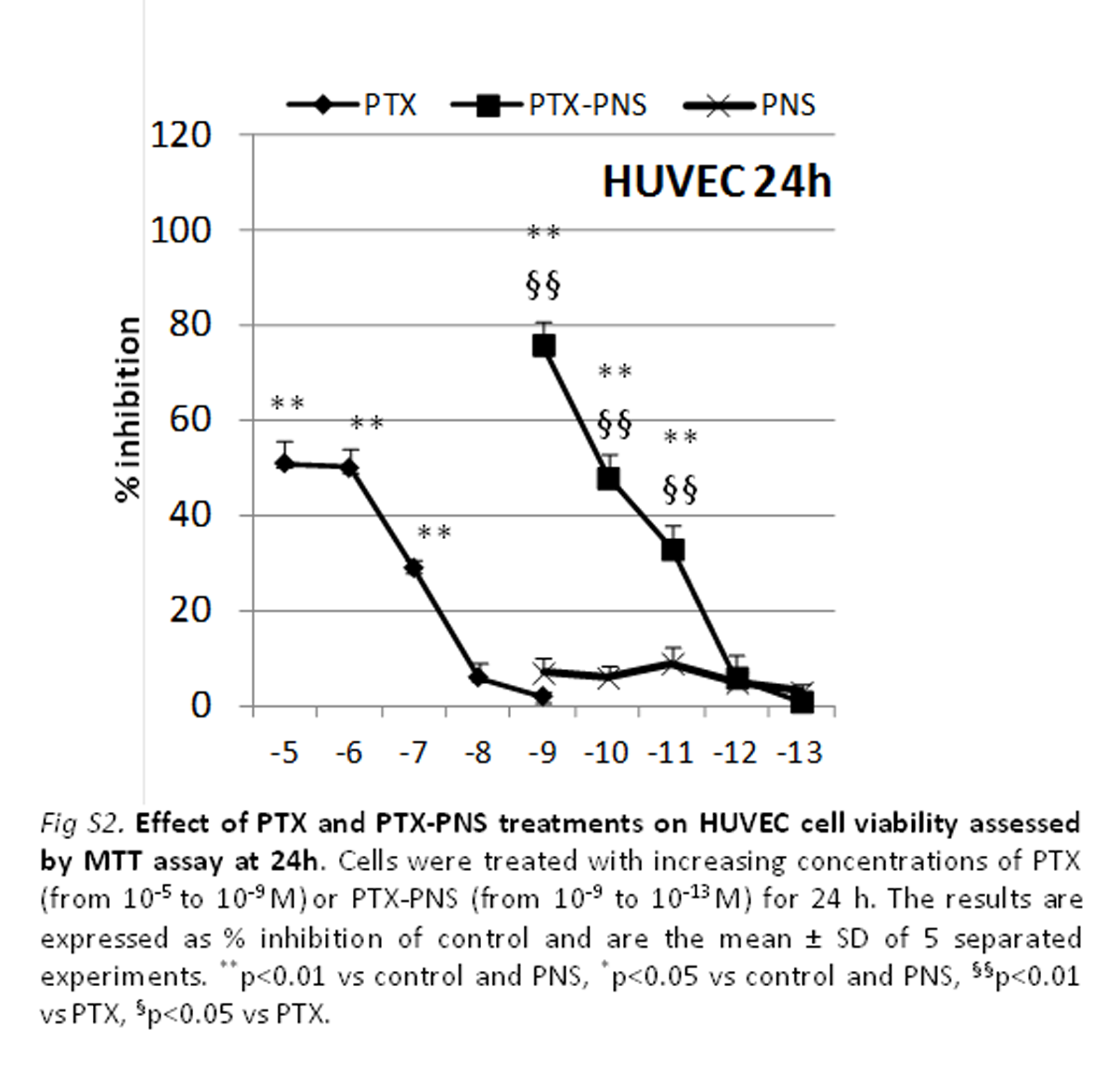

Supplement: Figure S2 — Effect of PTX and PTX-PNS treatments on HUVEC cell viability assessed by MTT assay at 24h. [file Image_2.tif]

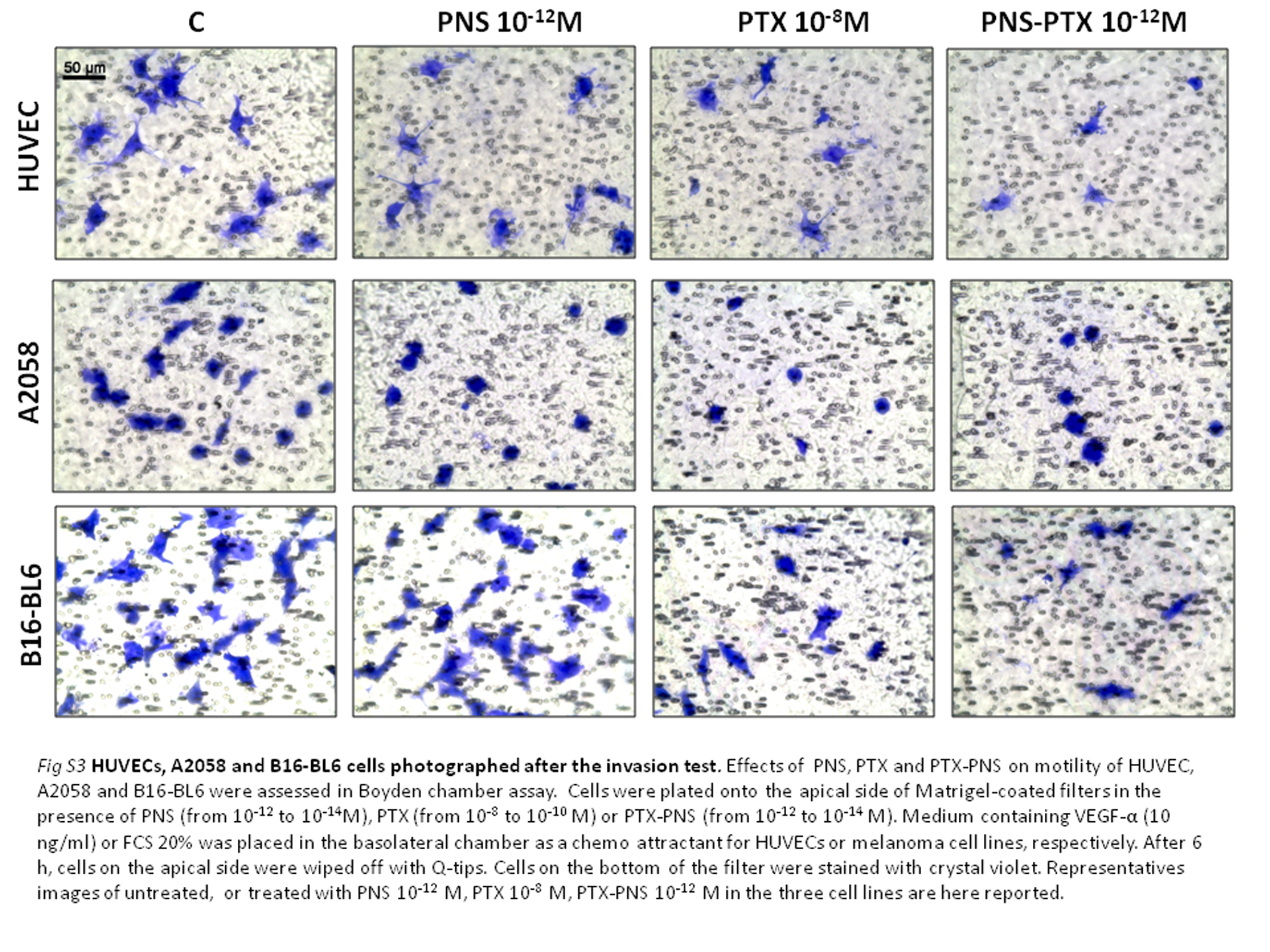

Supplement: Figure S3 — HUVECs, A2058 and B16-BL6 cells photographed after the invasion test. [file Image_3.tif]
