# Supplementary material for: Paclitaxel-Loaded Nanosponges Inhibit Growth and Angiogenesis in Melanoma Cell Models
Source: Front Pharmacol. 2019 Jul 12;10:776. doi: 10.3389/fphar.2019.00776 (PMC6639435; doi:10.3389/fphar.2019.00776)
Supplement: Table S1 — Effect of PTX and PTX-PNS treatments on HUVEC, A2058, and B16-BL6 on cell viability assessed by crystal violet staining at 6h. [file Table_1.docx]

Table S1. **Effect of PTX and PTX-PNS treatments on HUVEC, A2058, and B16-BL6 on cell viability assessed by crystal violet staining at 6h.** Cells were untreated (control) or treated with increasing concentrations of PTX (from 10^-7^ to 10^-9^ M) or PTX-PNS (from 10^-10^ to 10^-14^ M), and with PNS 10^-10^ M for 6 h. The controls were normalized to 100% and the readings from treated cells were expressed as % of viability inhibition. They are the mean ± SD of four replicates of 5 separated experiments.

|  | HUVEC | A2058 | B16-BL6 |
| --- | --- | --- | --- |
| PNS 10^-10^ M | 6.4 ± 3.6 | 5.3 ± 3.4 | 4.6 ± 3.9 |
| PTX 10^-7^ M | 15.1 ± 7.3 | 13.2 ± 7.4 | 10.6 ± 2.7 |
| PTX 10^-8^ M | 14.2 ± 7.4 | 12.7 ± 7.4 | 4.5 ± 2.4 |
| PTX 10^-9^ M | 8.8 ± 3.9 | 4.1 ± 3.7 | 2.9 ± 2.0 |
| PTX-PNS 10^-10^ M | 16.1 ± 5.1 | 14.5 ± 6.9 | 10.1 ± 3.2 |
| PTX-PNS 10^-11^ M | 12.6 ± 4.4 | 11.4 ± 3.6 | 8.7 ± 4.5 |
| PTX-PNS 10^-12^ M | 6.2 ± 3.4 | 5.7 ± 3.8 | 4.2 ± 4.7 |
| PTX-PNS 10^-13^ M | 8.8 ± 5.2 | 6.4 ± 4.6 | 7.2 ± 3.4 |
| PTX-PNS 10^-14^ M | 7.6 ± 4.7 | 6.5 ± 3.1 | 5.1 ± 4.4 |
